# Echocardiographic estimation of pulmonary vascular resistance in advanced lung disease

**DOI:** 10.1002/pul2.12183

**Published:** 2023-01-06

**Authors:** Jacqueline T. DesJardin, Yana Svetlichnaya, Nicholas A. Kolaitis, Steven R. Hays, Jasleen Kukreja, Nelson B. Schiller, Lucas S. Zier, Jonathan P. Singer, Teresa De Marco

**Affiliations:** ^1^ Division of Cardiology University of California San Francisco San Francisco California USA; ^2^ Division of Cardiology Kaiser Permanente San Francisco California USA; ^3^ Division of Pulmonary, Critical Care, Allergy, and Sleep Medicine University of California San Francisco San Francisco California USA; ^4^ Division of Adult Cardiothoracic Surgery University of California San Francisco San Francisco California USA; ^5^ Division of Cardiology Zuckerberg San Francisco General Hospital and Trauma Center San Francisco California USA

**Keywords:** chronic obstructive pulmonary disease, hemodynamics, interstitial lung disease, lung transplant, pulmonary hypertension

## Abstract

Noninvasive assessment of pulmonary hemodynamics is often performed by echocardiographic estimation of the pulmonary artery systolic pressure (ePASP), despite limitations in the advanced lung disease population. Other noninvasive hemodynamic variables, such as echocardiographic pulmonary vascular resistance (ePVR), have not been studied in this population. We performed a retrospective analysis of 147 advanced lung disease patients who received both echocardiography and right heart catheterization for lung transplant evaluation. The ePVR was estimated by four previously described equations. Noninvasive and invasive hemodynamic parameters were compared in terms of correlation, agreement, and accuracy. The ePVR models strongly correlated with invasively determined PVR and had good accuracy with biases of <1 Wood units (WU), although with moderate precision and wide 95% limits of agreement varying from 5.9 to 7.8 Wood units. The ePVR models were accurate to within 1.9 WU in over 75% of patients. In comparison to the ePASP, ePVR models performed similarly in terms of correlation, accuracy, and precision when estimating invasive hemodynamics. In screening for pulmonary hypertension, ePVR models had equivalent testing characteristics to the ePASP. Mid‐systolic notching of the right ventricular outflow tract Doppler signal identified a subgroup of 11 patients (7%) with significantly elevated PVR and mean pulmonary artery pressures without relying on the acquisition of a tricuspid regurgitation signal. Analysis of ePVR and determination of the notching pattern of the right ventricular outflow tract Doppler flow velocity envelope provide reliable insights into hemodynamics in advanced lung disease patients, although limitations in precision exist.

**Abbreviations**
cPVR: catheter‐derived pulmonary vascular resistance
ePASP: echocardiographic pulmonary artery systolic pressure
ePVR: echocardiographic pulmonary vascular resistance
FVE_RVOT_
: right ventricular outflow tract Doppler flow velocity envelope
LSN: late‐systolic notching of the VTI_RVOT_ Doppler signal
mPAP: mean pulmonary artery pressure
MSM: mid‐systolic notching of the VTI_RVOT_ Doppler signal
NN: no notching of the VTI_RVOT_ Doppler signal
PAWP: pulmonary arterial wedge pressure
PVR: pulmonary vascular resistance
RVOT: right ventricular outflow tract
TRV: tricuspid regurgitant peak velocity
VTI_RVOT_
: right ventricular outflow tract time velocity integral

## INTRODUCTION

Hemodynamic parameters have significant implications for prognosis, treatment, transplant eligibility, and transplant listing in patients with advanced lung disease.^1^, ^2^ In idiopathic pulmonary fibrosis, pulmonary hypertension is associated with a 4.6‐fold increase in 1‐year mortality, while in chronic obstructive pulmonary disease, pulmonary hemodynamics are stronger predictors of survival than lung function or gas exchange variables.^3^, ^4^, ^5^

Transthoracic echocardiography is the major noninvasive method used to screen advanced lung disease patients for pulmonary hypertension and monitor disease progression. Echocardiographic screening for pulmonary hypertension is usually performed by estimating the pulmonary artery systolic pressure (ePASP) from the tricuspid regurgitant peak velocity (TRV). Despite its common clinical use, the ePASP has several limitations. First, due to increased difficulty in acquiring tricuspid regurgitant signals, the ePASP cannot be estimated in many advanced lung disease patients.^6^, ^7^ Even when the TRV is adequate for the determination of the ePASP, there remains controversy over the reliability of this measurement. Several prior studies have reported weak correlation and limited precision between echocardiographic and invasively measured PASP in advanced lung disease patients.^8^, ^9^, ^10^, ^11^, ^12^ In lung transplant candidates, up to half of ePASP measurements are inaccurate by over 10 mmHg,^7^, ^13^ and 17%–28% of measurements are incorrect by over 20 mmHg.^7^, ^10^ There is a need to validate noninvasive parameters that can reliably estimate hemodynamics, screen for pulmonary hypertension, and predict outcomes in advanced lung disease patients.

Several methods for echocardiographic estimation of pulmonary vascular resistance (ePVR) have been developed, but they have not been well‐validated in advanced lung disease.^14^, ^15^, ^16^ Unlike ePASP, ePVR differentiates precapillary from postcapillary pulmonary hypertension and has been shown to improve pulmonary hypertension screening in liver transplant candidates.^14^, ^15^, ^16^, ^17^, ^18^ Estimation of ePVR has particular relevance for advanced lung disease patients, in whom prognosis and candidacy for inhaled treprostinil are both closely tied to PVR.^2^, ^4^, ^19^, ^20^, ^21^ Additionally, interpretation of the shape of the right ventricular outflow tract (RVOT) Doppler flow velocity envelope (FVE_RVOT_) reliably predicts high PVR in other patient populations and has the advantage of not requiring a measurable tricuspid regurgitant jet.^15^

In this study, we sought to validate equations for ePVR estimation in advanced lung disease patients against invasive, catheter‐derived PVR (cPVR). We examined the correlation, agreement, accuracy, and pulmonary hypertension screening characteristics for ePVR models and compared them to the ePASP. We further assessed the validity of using the shape of the FVE_RVOT_ for predicting invasive hemodynamics in this population.

## METHODS

### Patient selection

**Figure 1:**
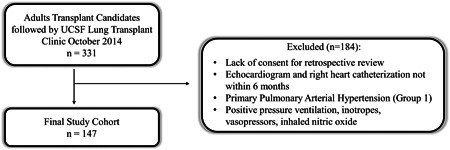
Consort diagram. UCSF, University of California San Francisco.

The study cohort was derived from adult patients followed by the lung transplantation clinic at the University of California San Francisco in October 2014. We included lung transplant candidates because, at our institution, all lung transplant candidates receive both transthoracic echocardiogram and right heart catheterization as part of the standard prelisting evaluation. Patients were included if they provided consent for retrospective medical record review and underwent echocardiography and catheterization within 6 months of each other. Patients were excluded if they had a prior diagnosis of pulmonary arterial hypertension (Group 1 pulmonary hypertension) or were receiving positive pressure ventilation, intravenous inotropes, vasopressors, and/or inhaled nitric oxide. The final study cohort was comprised of 147 patients (Figure 1). The median time between echocardiography and right heart catheterization was 12 (interquartile range: 2–44) days and 20% had the two procedures within 24 h of each other. This study was approved by the Institutional Review Board of the University of California San Francisco.

### Right heart catheterization

Right heart catheterization was performed using 5‐ or 7‐French balloon‐tipped catheters by experienced interventional or advanced heart failure cardiologists at our institution. Right heart catheterizations were performed at rest and without sedation or supplemental oxygen when feasible. Operators at our institution perform zeroing at the mid‐thoracic line halfway between the bed and the anterior sternum with the patient in a supine position as previously described.^22^ The following pressure measurements were obtained at end‐expiration (functional residual capacity): catheter‐derived right atrial pressure, right ventricular pressure, pulmonary artery systolic pressure (cPASP), pulmonary artery end‐diastolic pressure, mean pulmonary artery pressure (mPAP), and pulmonary arterial wedge pressure (PAWP). To avoid inaccuracies due to pronounced respiratory variation, right heart and pulmonary pressures were averaged over multiple cardiac and respiratory cycles. Pulmonary artery wedge saturations are also frequently obtained at our institution to confirm occlusive PAWP in patients undergoing catheterization for the purpose of heart or lung transplant listing. A left‐ventricular end‐diastolic pressure was used when a reliable PAWP tracing could not be obtained (*n* = 7). Cardiac output (CO) and cardiac index were calculated using the indirect Fick method. The indirect Fick method (rather than the thermodilution method) was used because this study occurred while our institution was transitioning from internal jugular to brachial venous access, and not all catheters had thermodilution capability. cPVR was calculated using the formula: cPVR (Wood units [WU]) = (mPAP − PAWP)/CO.

### Transthoracic echocardiography

All subjects underwent conventional M‐mode, two‐dimensional, and color Doppler imaging using commercially available ultrasound systems (Vingmed Ultrasound, Vivid 7GE Healthcare or Phillips Sonos, Philips Medical Systems). Pulsed‐wave Doppler interrogation of the RVOT was performed in the short‐axis parasternal view. Measurements included the peak velocity of the tricuspid regurgitant jet (TRV, m/s) and RVOT velocity time integral (VTI_RVOT_). Interrogation of the tricuspid valve was performed in multiple views to find the most complete Doppler envelope with the highest TRV. Agitated saline was administered at the discretion of the sonographer to enhance the tricuspid regurgitant jet. At least three cardiac cycles were measured for TRV and VTI_RVOT_ and average values were used.

A single reader blinded to the results of invasive hemodynamics (J. P. S.) interpreted all studies and categorized the shape of FVE_RVOT_ as no notching (NN), late‐systolic notching (LSN), or mid‐systolic notching (MSN). MSN was defined by a notch (distinct flow velocity deceleration) within the initial two‐thirds of the systolic ejection period, while LSN was a notch in the terminal one‐third of the Doppler signal as previously described.^15^ All tracings were re‐examined by a second reader for interobserver variability. The shape of the FVE_RVOT_ was interpretable in all patients and there was substantial agreement in the identification of MSN between the two readers (97% agreement, *κ* = 0.82). The ePASP and four previously described echocardiographic equations for noninvasive estimation of PVR (termed ePVR1, ePVR2, ePVR3, and ePVR4) were calculated for each patient using Doppler‐derived variables (Table 1).^14^, ^15^, ^16^, ^23^

**Table 1:** Noninvasive models for estimation of pulmonary artery systolic pressure and pulmonary vascular resistance

Model and reference	Equation
ePASP (Rudski et al.^23^: American Society of Echocardiography Guidelines)	4 × TRV^2^ + eRAP
ePVR1 (Abbas et al.^14^)	TRV/VTI_RVOT_ × 10 + 0.16
ePVR2 (Abbas et al.,^16^ simplified version)	If TRV/VTI_RVOT_ > 0.275: TRV^2^/VTI_RVOT_ × 5
	If TRV/VTI_RVOT_ ≤ 0.275: TRV/VTI_RVOT_ × 10
ePVR3 (Opotowsky et al.^24^)	ePASP/VTI_RVOT_ × 1.2
ePVR4 (Opotowsky et al.^24^)	ePASP/VTI_RVOT_ + 3 if MSN present

Abbreviations: ePASP, echocardiographic pulmonary artery systolic pressure; ePVR, echocardiographic pulmonary vascular resistance; eRAP, echocardiographic right atrial pressure; MSN, mid‐systolic notching of the right ventricular outflow tract Doppler flow velocity envelope; TRV, tricuspid regurgitant peak velocity; VTIRVOT, right ventricular outflow tract velocity time integral.

### Statistical analysis

Continuous values are expressed as mean ± standard deviation or median with interquartile range, as appropriate. Categorical values are expressed as absolute and relative frequencies. Pearson's correlation and Bland–Altman analysis were used to assess correlation and agreement between invasive and noninvasive measurements. Accuracy was described by displaying the percentage of ePVR or ePASP values, which fell within 1 SD of the invasively measured variable. Receiver‐operator characteristics curves were generated to examine the testing characteristics of the ePVR models for the prediction of pulmonary hypertension. The ePVR models were compared to the TRV, which is the recommended echocardiographic method for the estimation of pulmonary hypertension by current guidelines.^25^ Pulmonary hypertension was defined as mPAP > 20 mmHg on right heart catheterization and precapillary pulmonary hypertension was defined as mPAP > 20 mmHg, PAWP ≤ 15 mmHg, and PVR > 2 WUs, as is consistent with current guidelines.^25^ Differences in mPAP and cPVR were assessed by echocardiographic FVE_RVOT_ notching pattern (i.e., NN, MSN, LSN) using Wilcoxon's rank‐sum tests because the hemodynamic measurements were not normally distributed. Normalcy was determined by graphical methods (plotting histograms of data) as well as quantitative methods (Shapiro–Wilk test for normality). Sensitivity analysis was performed including only patients who underwent right heart catheterization and echocardiography within 4 weeks of each other (*n* = 98). Stata version 15.1 was used for all statistical analyses; *p* < 0.05 was considered statistically significant.

## RESULTS

The study cohort was comprised of 147 LT candidates; 49% of patients had idiopathic pulmonary fibrosis, 25% had chronic obstructive pulmonary disease, and 8% had cystic fibrosis. The baseline characteristics of the study population are shown in Table 2. Pulmonary hypertension was present in 61% of the cohort, and the majority of pulmonary hypertension was precapillary.

**Table 2:** Characteristics of study participants

	Total cohort (*n* = 147)
Age, mean (SD) (years)	55.7 (11.5)
Sex, *n* (%*N*)	
Male	89 (61%)
Female	58 (39%)
Lung Transplant Allocation Group, *n* (%*N*)	
A	37 (25%)
C	12 (8%)
D	98 (67%)
Lung disease, *n* (%*N*)	
Idiopathic pulmonary fibrosis	72 (49%)
Chronic obstructive pulmonary disease	37 (25%)
Cystic fibrosis	12 (8%)
Other	26 (18%)
Hemodynamics on right heart catheterization, mean (SD)	
Right atrial pressure (mmHg)	4.5 (3.1)
mPAP (mmHg)	24.5 (9.4)
cPASP (mmHg)	38.8 (14.4)
cPVR (WU)	3.4 (1.9)
PAWP (mmHg)	8.3 (4.2)
Indirect Fick cardiac output (L/min)	5.1 (1.3)
Indirect Fick cardiac index (L/min/m^2^)	2.8 (0.6)
Stroke volume index (ml/m^2^)	36.9 (10.3)
Echocardiographic parameters	
TRV (m/s) (*n* = 133)	3.1 (0.7)
ePASP (mmHg) (*n* = 133)	43.1 (19.2)
ePVR1 (WU) (*n* = 133)	2.6 (0.9)
ePVR2 (WU) (*n* = 133)	3.4 (2.8)
ePVR3 (WU) (*n* = 133)	4.1 (2.5)
ePVR4 (WU) (*n* = 133)	3.7 (2.6)
TAPSE (cm) (*n* = 84)	2.0 (0.4)
TAPSE/ePASP (mm/mmHg) (*n* = 72)	0.56 (0.25)
Pulmonary hypertension, *n* (%N)	
Pulmonary hypertension	89 (60.5%)
Precapillary pulmonary hypertension	74 (50.3%)

*Note*: Pulmonary hypertension = mPAP > 20 mmHg on right heart catheterization. Precapillary pulmonary hypertension = mPAP > 20 mmHg, cPVR > 2 WU, and PAWP < 15 mmHg on right heart catheterization.

Abbreviations: cPVR, catheter‐derived pulmonary vascular resistance; ePASP, echocardiographic pulmonary artery systolic pressure; ePVR1, echocardiographic pulmonary vascular resistance Model 1; ePVR2, echocardiographic pulmonary vascular resistance Model 2; ePVR3, echocardiographic pulmonary vascular resistance Model 3; ePVR4, echocardiographic pulmonary vascular resistance Model 4; mPAP, mean pulmonary artery pressure; PASP, pulmonary artery systolic pressure; PAWP, pulmonary arterial wedge pressure; SD, standard deviation; TAPSE, tricuspid annular plane systolic excursion; TRV, tricuspid regurgitation velocity.

**Figure 2:**
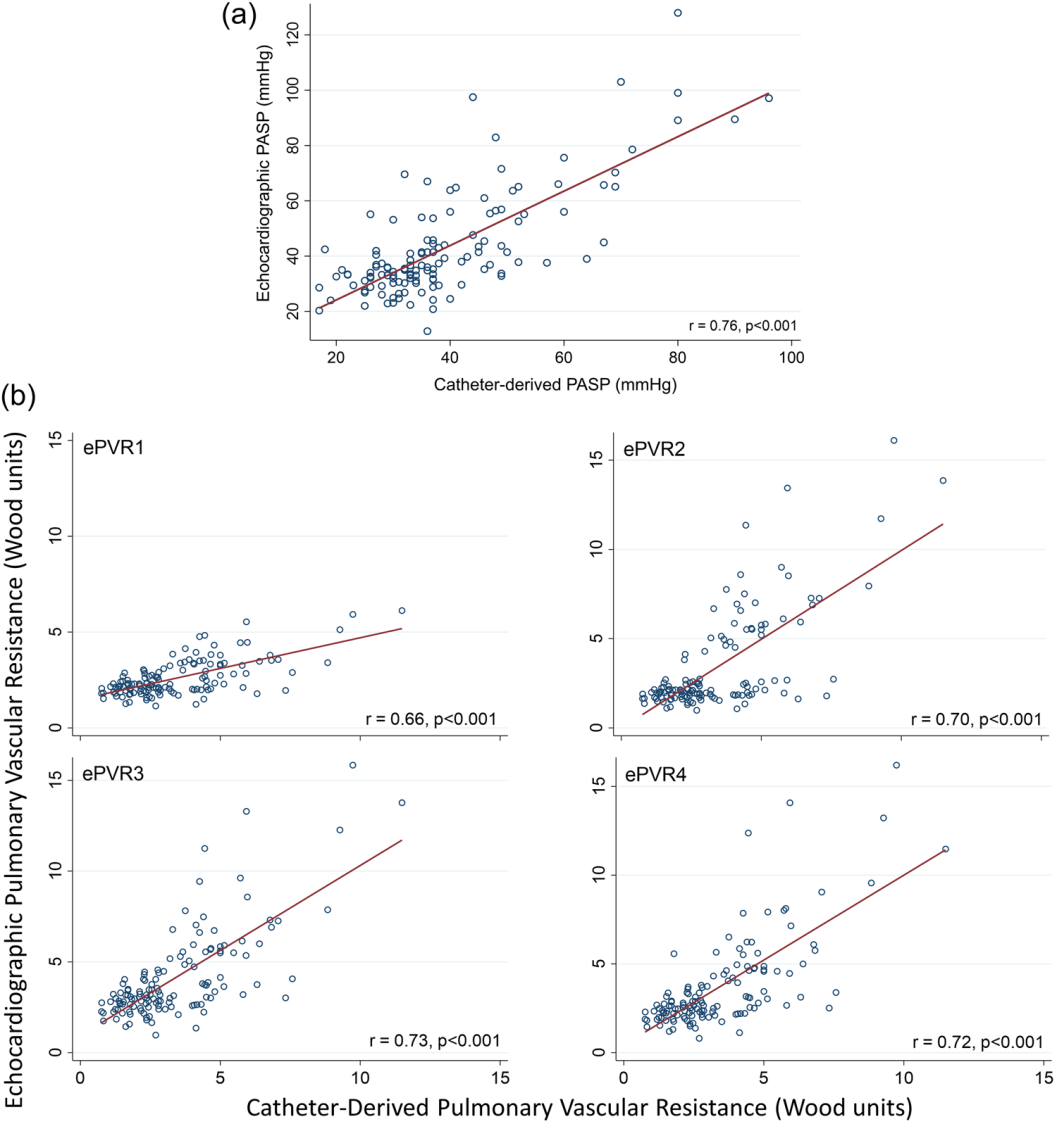
Correlation between echocardiographic and invasive parameters. (a) Systolic pulmonary artery pressure estimated by echocardiography versus measured by right heart catheterization. (b) Pulmonary vascular resistance was estimated by echocardiography versus measured by right heart catheterization. ePVR1, echocardiographic pulmonary vascular resistance Model 1; ePVR2, echocardiographic pulmonary vascular resistance Model 2; ePVR3, echocardiographic pulmonary vascular resistance Model 3; ePVR4, echocardiographic pulmonary vascular resistance Model 4; PASP, pulmonary artery systolic pressure.

For the entire cohort, a tricuspid regurgitant jet of measurable quality to estimate the TRV, ePASP, and ePVR was observed in 133 patients (90%). Among the 14 patients without an interpretable TR jet, seven had pulmonary hypertension. There was a correlation between cPASP and ePASP (Figure 2a), as well as between cPVR and each of the ePVR models (Figure 2b).

**Figure 3:**
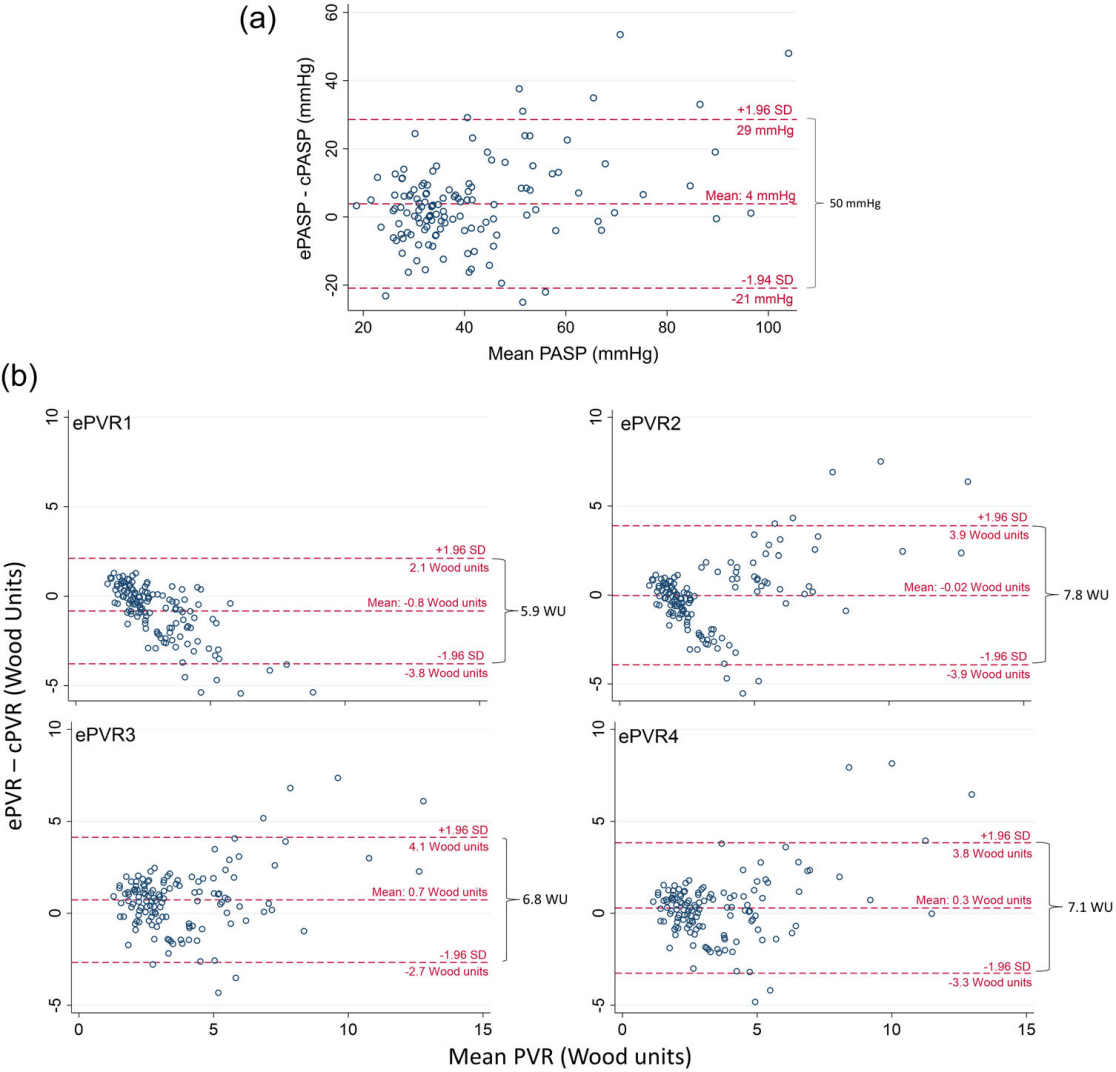
Bland–Altman analysis of echocardiographic parameters. (a) Pulmonary artery systolic pressure is estimated by echocardiography (ePASP) versus measured by right heart catheterization (cPASP). (b) Pulmonary vascular resistance estimated by echocardiography versus measured by right heart catheterization (cPVR). cPVR, catheter‐derived pulmonary vascular resistance; cPASP, catheter pulmonary artery systolic pressure; ePASP, echocardiographic pulmonary artery systolic pressure; ePVR1, echocardiographic pulmonary vascular resistance Model 1; ePVR2, echocardiographic pulmonary vascular resistance Model 2; ePVR3, echocardiographic pulmonary vascular resistance Model 3; ePVR4, echocardiographic pulmonary vascular resistance Model 4.

**Figure 4:**
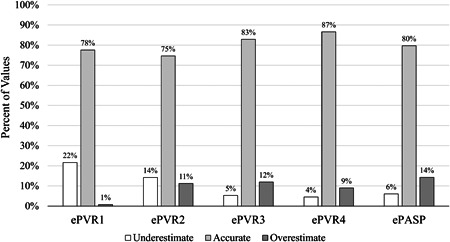
Accuracy of echocardiographic parameters. Echocardiographic pulmonary vascular resistance models were considered accurate if they fell within 1.9 WU [1 SD] of the catheter‐derived PVR. ePASP was considered accurate if it fell within 14.4 mmHg [1 SD] of the catheter‐derived PASP. ePASP, echocardiographic pulmonary artery systolic pressure; ePVR1, echocardiographic pulmonary vascular resistance Model 1; ePVR2, echocardiographic pulmonary vascular resistance Model 2; ePVR3, echocardiographic pulmonary vascular resistance Model 3; ePVR4, echocardiographic pulmonary vascular resistance Model 4.

**Figure 5:**
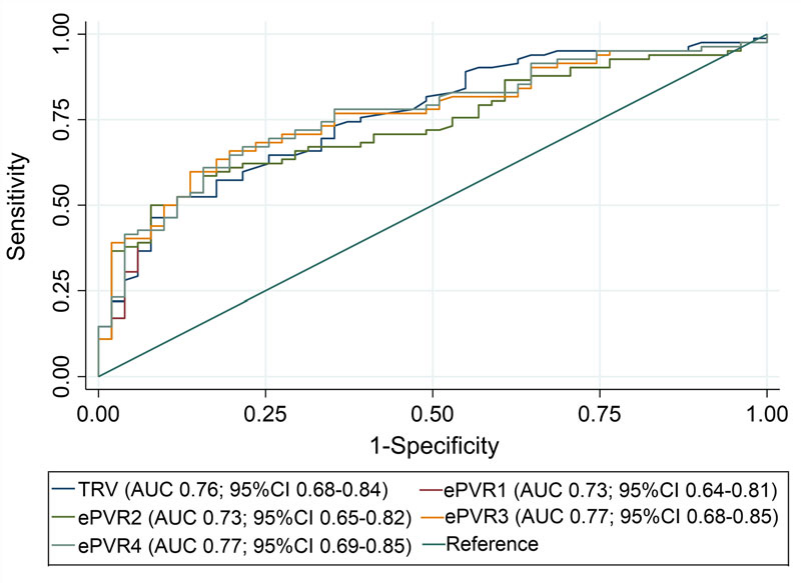
Receiver‐operating characteristic curves for echocardiographic pulmonary vascular resistance and tricuspid regurgitant velocity in discrimination of pulmonary hypertension. AUC, area under the receiver‐operating characteristics curve. ePVR, echocardiographic pulmonary vascular resistance models; ePVR1, echocardiographic pulmonary vascular resistance Model 1; ePVR2, echocardiographic pulmonary vascular resistance Model 2; ePVR3, echocardiographic pulmonary vascular resistance Model 3; ePVR4, echocardiographic pulmonary vascular resistance Model 4; TRV, tricuspid regurgitant velocity.

**Figure 6:**
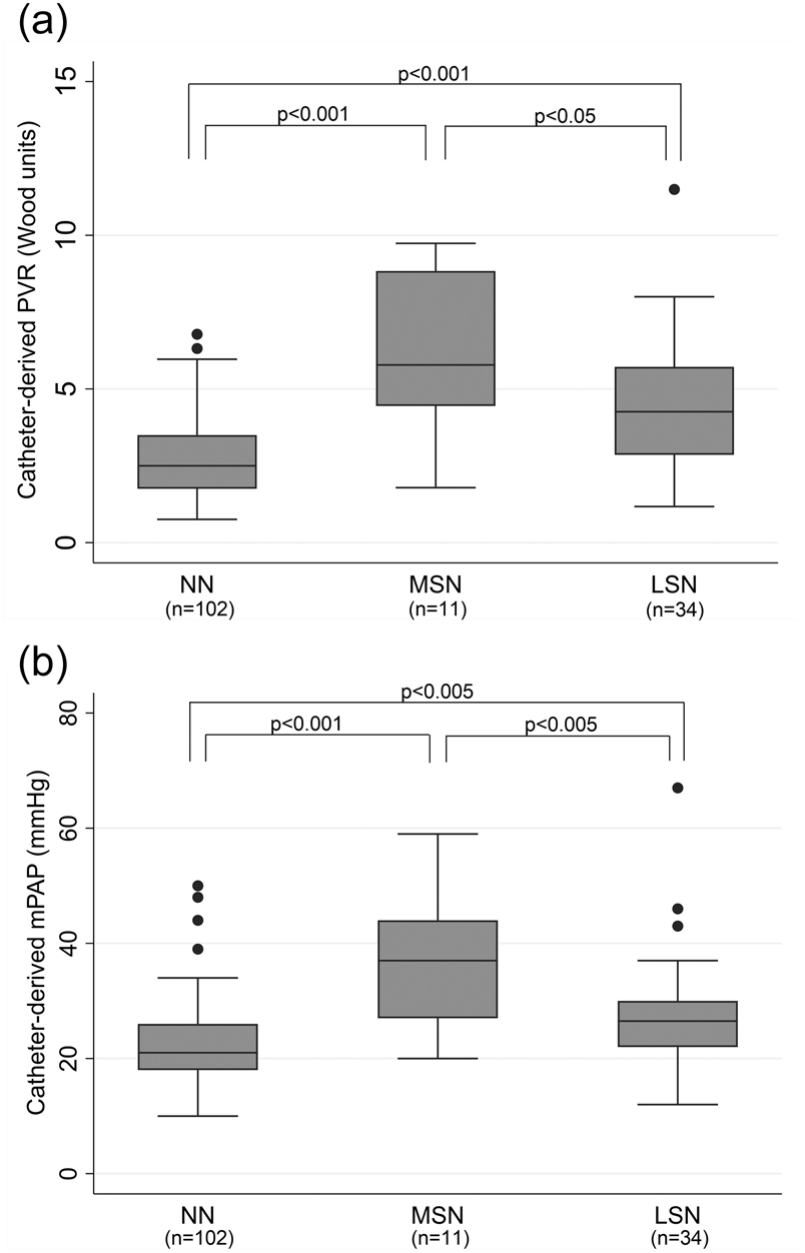
Hemodynamic differences by right ventricular outflow tract Doppler flow velocity envelope notching pattern. (a) Changes in PVR notching pattern. (b) Changes in mPAP. LSN, late‐systolic notching shape of the right ventricular outflow tract (RVOT) Doppler flow velocity envelope; mPAP, mean pulmonary artery pressure by notching pattern; MSN = mid‐systolic notching; NN, no notching; PVR = pulmonary vascular resistance; mPAP = mean pulmonary artery pressure.

Bland–Altman analysis of ePASP and cPASP revealed a bias of 4 mmHg with 95% limits of agreement ranging from ‐21 to 29 mmHg or a 50 mmHg range (Figure 3a). Bland–Altman analysis of cPVR and the ePVR equations revealed biases ranging from −0.8 (ePVR1) to 0.7 (ePVR3) WU, with the range of the 95% limits of agreement varying from 5.9 (ePVR1) to 7.8 (ePVR2) WU (Figure 3b). For ePVR1, the absolute difference between ePVR1 and cPVR decreased with increasing mean PVR such that at higher PVR measurements the ePVR1 method is increasingly likely to underestimate the true PVR (Figure 3b). Comparatively, for the ePASP and the other ePVR models, there is a trend in the opposite direction such that at high values echocardiographic metrics are more likely to overestimate true values (Figure 3a,b). When the TRV was measurable (90% of subjects, *n* = 133), the ePVR models were accurate (falling within 1.9 WU [1 SD] of cPVR) in over 75% of cases (Figure 4). The highest absolute accuracy was 87% in the ePVR4 model, although there were no significant differences in accuracy between ePVR models or the ePASP. Comparatively, when measurable, the ePASP was accurate (falling within 14.4 mmHg [1 SD] of cPASP) in 80% of cases (Figure 4). The ePVR models and TRV had similar testing characteristics for the prediction of pulmonary hypertension and there were no statistically significant differences in the area under receiver‐operating characteristics curves between various models (Figure 5). Unlike the TRV, the shape of the FVE_RVOT_ was interpretable in all 147 patients. The presence of MSN had 98% specificity in screening for pulmonary hypertension (Supporting Information: Table 1). MSN identified a subset of 11 patients with significantly elevated cPVR (Figure 6a) and mPAP (Figure 6b).

Sensitivity analysis including only patients who underwent echocardiography and right heart catheterization within 4 weeks of one another was performed (*n* = 97). For sensitivity analysis, the median time between echocardiography and right heart catheterization was 4 (Interquartile range: 1–12) days and 30% had the two procedures within 24 h of each other. The primary results were not substantially changed on sensitivity analysis (Supporting Information: Figures 1–6).

## DISCUSSION

In a cohort of advanced lung disease patients, echocardiographic estimation of PVR was found to be accurate, but with only moderate precision. The estimation of the PVR by echocardiography had similar accuracy and precision as the estimation of the PASP by echocardiography. In screening for pulmonary hypertension, the ePVR models and TRV had similar testing characteristics. Unlike the ePASP and ePVR estimates, the notching pattern of the FVE_RVOT_ was obtainable in all patients. MSN had high specificity for the prediction of pulmonary hypertension and identified a subgroup of patients with significantly elevated cPVR and mPAP.

Prior studies validating the use of Doppler echocardiography in the assessment of pulmonary hypertension in advanced lung disease have relied heavily on correlation calculations.^7^, ^9^ As discussed by Bland and Altman, the use of correlation is misleading as two methods for measuring the same thing can often strongly correlate but not agree, especially when one method systematically under‐ or overestimates the true value.^26^, ^27^ Assessment of measurement error necessitates the use of Bland–Altman analysis, which provides a graphical representation of both accuracy (e.g., bias) and precision (e.g., 95% limits of agreement). Prior studies employing Bland–Altman analysis have also found that echocardiographic measurements such as the ePASP have limitations in precision.^28^ In patients with lung disease, studies have shown good accuracy with bias <7 mmHg, but with limitations in precision and 95% limits of agreement ranging from −19 to −45 and +24 to +54 mmHg.^9^, ^11^, ^12^

Our study was one of the largest to examine echocardiographic parameters in lung transplant candidates, and the first to directly compare four well‐validated ePVR equations in the advanced lung disease population. We assessed whether ePVR models might have comparable agreement and accuracy to the commonly used ePASP. Overall, the ePVR models performed similarly, with the exception of ePVR1 which was the only model which progressively underestimated the true PVR at high values. Due to lack of precision, the use of echocardiography to estimate pulmonary hemodynamics may be valid for population studies but cannot be used for diagnosis of pulmonary hypertension in individual patients—a concept that is extensively covered in D'Alto et al.^28^ and further validated in this population by the present study. As with ePASP, ePVR has notable limitations in precision, particularly at extreme values. However, ePVR had correlation, accuracy, precision, and discrimination which were comparable to the ePASP. Currently, the ePASP is used routinely in clinical practice in this patient population, and from the present analysis, one could be assured that models of ePVR would perform similarly in estimating the true PVR. Therefore, estimation of PVR by echocardiography can be performed in advanced lung disease if clinicians understand the limitations that are inherent to estimating pulmonary hemodynamics by Doppler echocardiography. While echocardiography does not have sufficient precision to replace right heart catheterization, incorporation of ePVR into the echocardiographic assessment of advanced lung disease patients could help clinicians follow patients over time or noninvasively differentiate pulmonary vascular disease from elevated pulmonary pressures due to venous congestion and/or high flow. Therefore, this noninvasive tool may help clinicians identify patients who develop elevated PVR and might benefit from referral for catheterization and consideration of pulmonary vasodilators.^2^

We also described the benefits of assessment of the FVE_RVOT_ notching pattern in advanced lung disease patients. Determination of the ePASP and ePVR is limited to patients with tricuspid regurgitant jets of adequate quality to estimate the TRV. Older studies have suggested that the TRV cannot be determined in up to half of lung transplant candidates, possibly due to increased residual volume and changes in the positioning of the heart.^6^, ^29^ However, more recent studies have been able to recover sufficient echocardiographic signals in ~90% of patients with lung disease.^12^, ^30^ Similarly, in our study TRV could not be determined in 10% of patients, despite these patients still being at significant risk of pulmonary hypertension. Evaluation of the FVE_RVOT_ notching pattern does not rely on the TRV and was able to be determined in all patients in this cohort with high interrater reliability. MSN of the FVE_RVOT_ identified a subset of patients who had markedly elevated cPVR and mPAP. Although only identifiable in a subset of patients, when present, MSN reliably detects pulmonary hypertension in this population. The presence of MSN is therefore a dependable indicator of pulmonary vascular disease and pulmonary hypertension in lung transplant candidates and may be particularly useful when evaluating patients without an identifiable TRV. Further research should investigate the utility of other echocardiographic measures, such as the pulmonary artery acceleration time, in predicting pulmonary hemodynamics in advanced lung disease patients.^31^

Limitations of this study include the retrospective design, the use of a single‐institution sample, and limited available data on nonpulmonary comorbidities. Although right heart catheterization is the universally recognized gold standard for the assessment of pulmonary hemodynamics, the use of fluid‐filled catheters with insufficient frequency response, practice variations in technique (e.g., zeroing), and measurement error introduce inherent bias and limit reproducibility.^22^, ^28^, ^32^ These limitations are particularly pronounced in patients with advanced lung disease, whose pulmonary hemodynamics vary considerably during the respiratory cycle.^22^ An additional limitation is that echocardiography and catheterization hemodynamic data were not collected simultaneously. While awaiting transplantation, the pulmonary vascular disease of advanced lung disease patients worsens at an average of 3.8 mmHg/month.^33^ Although the median time between catheterization and echocardiography was only 12 days in this study, there is a risk that pulmonary hemodynamics progressed during this period. However, sensitivity analysis including only patients who underwent echocardiography and catheterization within 4 weeks of each other showed findings that were not substantially different from the main findings. Additionally, if hemodynamics significantly worsened between these studies, we would expect the ePASP and ePVR to systematically underestimate the severity of the pulmonary vascular disease. As with prior studies, we found that both ePASP and, to a lesser extent, ePVR have a systematic bias towards overestimation, suggesting that the progression of pulmonary vascular disease was not the main driver of discrepancies between noninvasive and invasive measurements. Systematic overestimation of ePASP and ePVR in ALD patients most likely occurs from not assigning peak TRV at the modal frequency.^6^

Analysis of ePVR and FVE_RVOT_ notching are accurate indicators of hemodynamics in advanced lung disease but lack precision. Calculation of ePVR and consideration of the FVE_RVOT_ shape may have utility in this population, and these assessments should be performed in advanced lung disease patients receiving echocardiography. Further research with a larger multicenter sample should confirm the described findings, as well as assess if these noninvasive parameters can predict outcomes in advanced lung disease patients.

## AUTHOR CONTRIBUTIONS

Yana Svetlichnaya, Jonathan P. Singer, and Teresa De Marco conceived and designed the study. Yana Svetlichnaya performed data collection. Jacqueline T. DesJardin performed data analysis. Jacqueline T. DesJardin, Yana Svetlichnaya, Nicholas A. Kolaitis, Jonathan P. Singer, and Teresa De Marco participated in data interpretation and presentation. Jacqueline T. DesJardin wrote the manuscript. All authors revised the manuscript.

## CONFLICTS OF INTEREST

Nicholas A. Kolaitis reports receiving consulting fees from United Therapeutics and advisory board fees for United Therapeutics and Bayer. Jonathan P. Singer reports serving on the advisory board of Altavant Sciences. Teresa De Marco reports consulting fees from Actelion, United Therapeutics, Arena, and SCOPE/Bial, and receives research funding from Acceleron.

## ETHICS STATEMENT

Approved by the Institutional Review Board of the University of California San Francisco, all patients consented to participation.

## Supporting information

Supporting information

Supporting information

Supporting information

Supporting information

Supporting information

Supporting information

Supporting information

Supporting information

Supporting information
