# Supplementary figures and images for: Echocardiographic estimation of pulmonary vascular resistance in advanced lung disease
Source: Pulm Circ. 2023 Jan 6;13(1):e12183. doi: 10.1002/pul2.12183 (PMC9817072; doi:10.1002/pul2.12183)

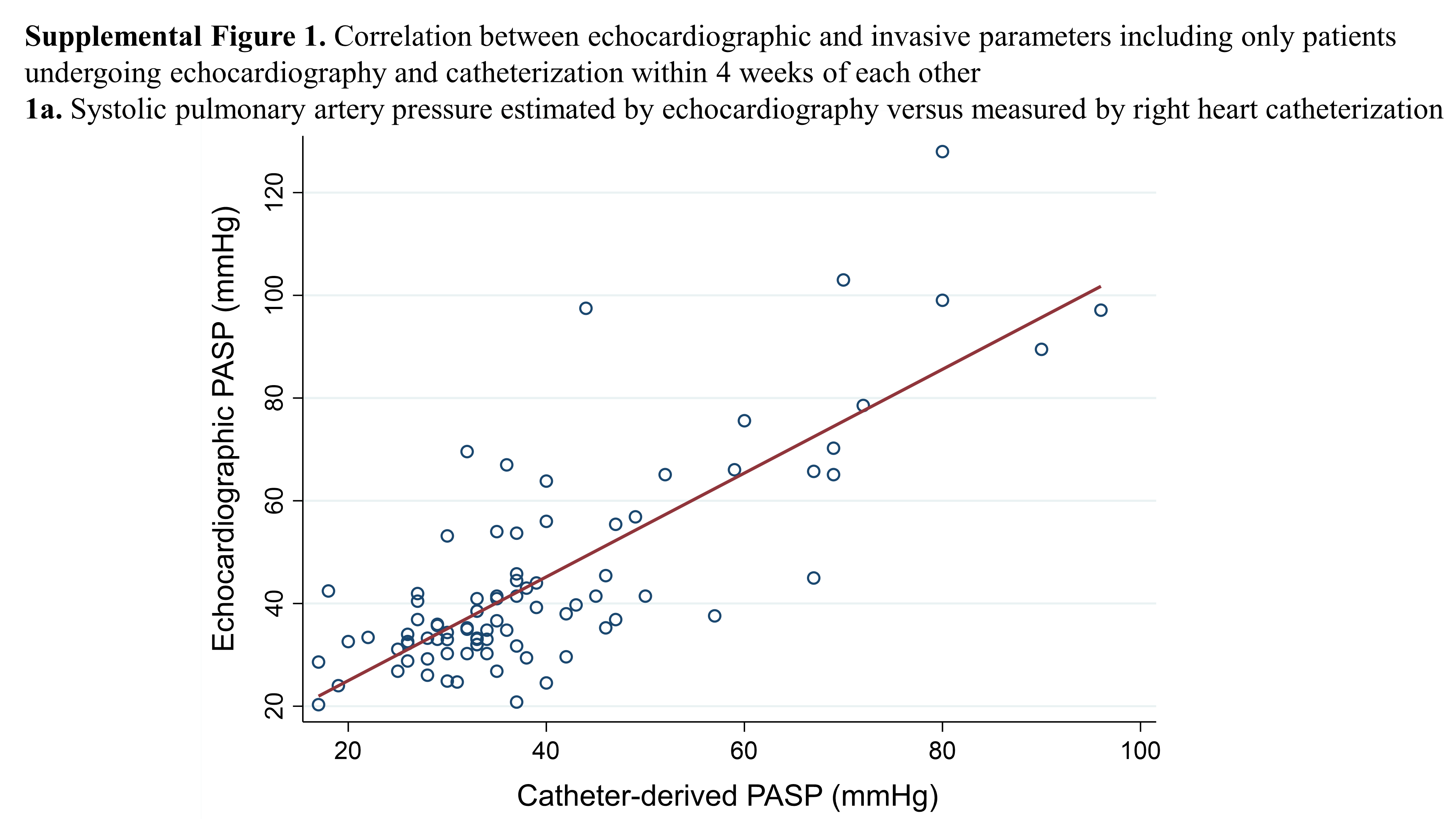

Supplement: Supplementary file 1 — Supporting information [file PUL2-13-e12183-s002.tif]

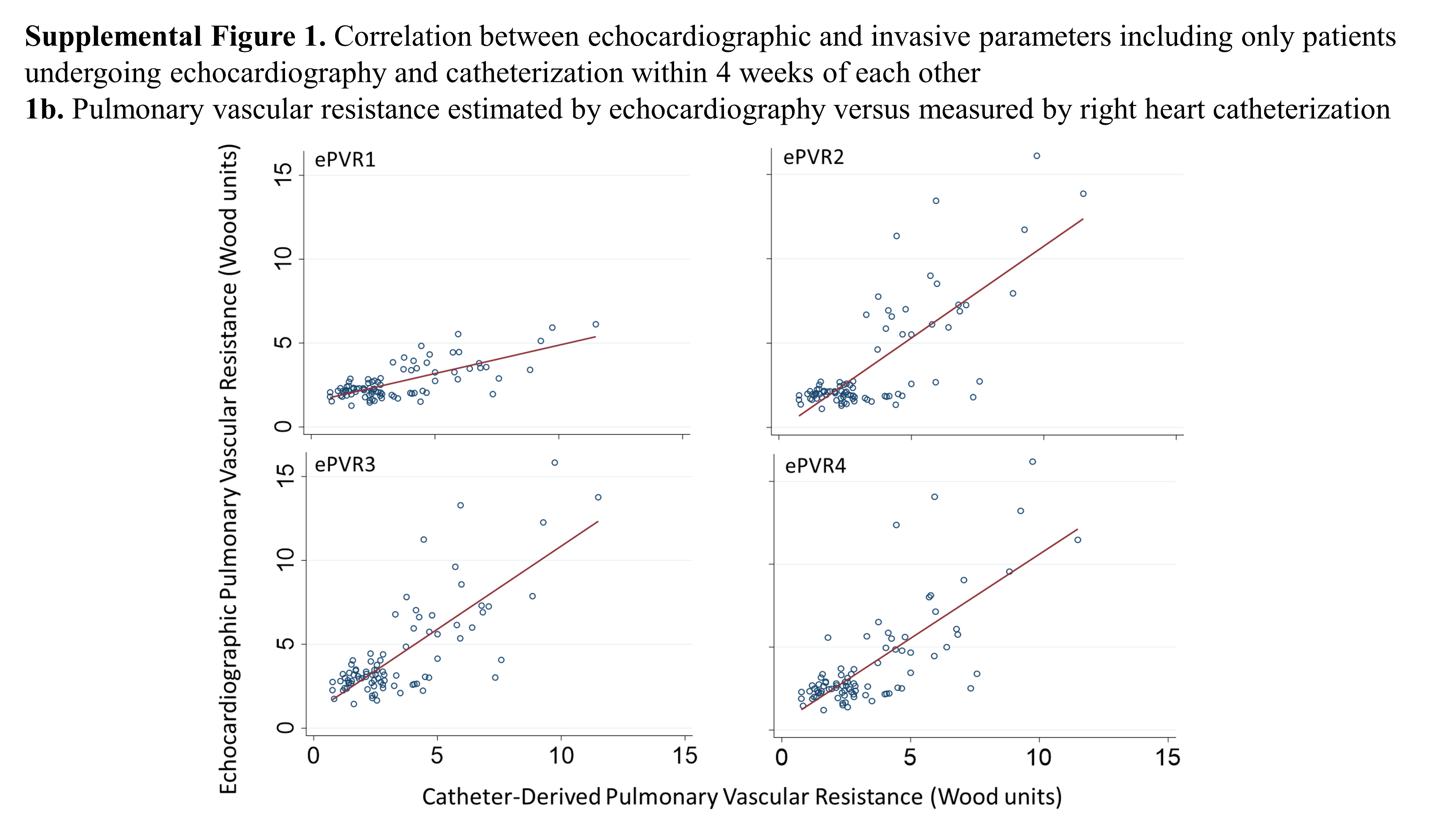

Supplement: Supplementary file 2 — Supporting information [file PUL2-13-e12183-s004.tif]

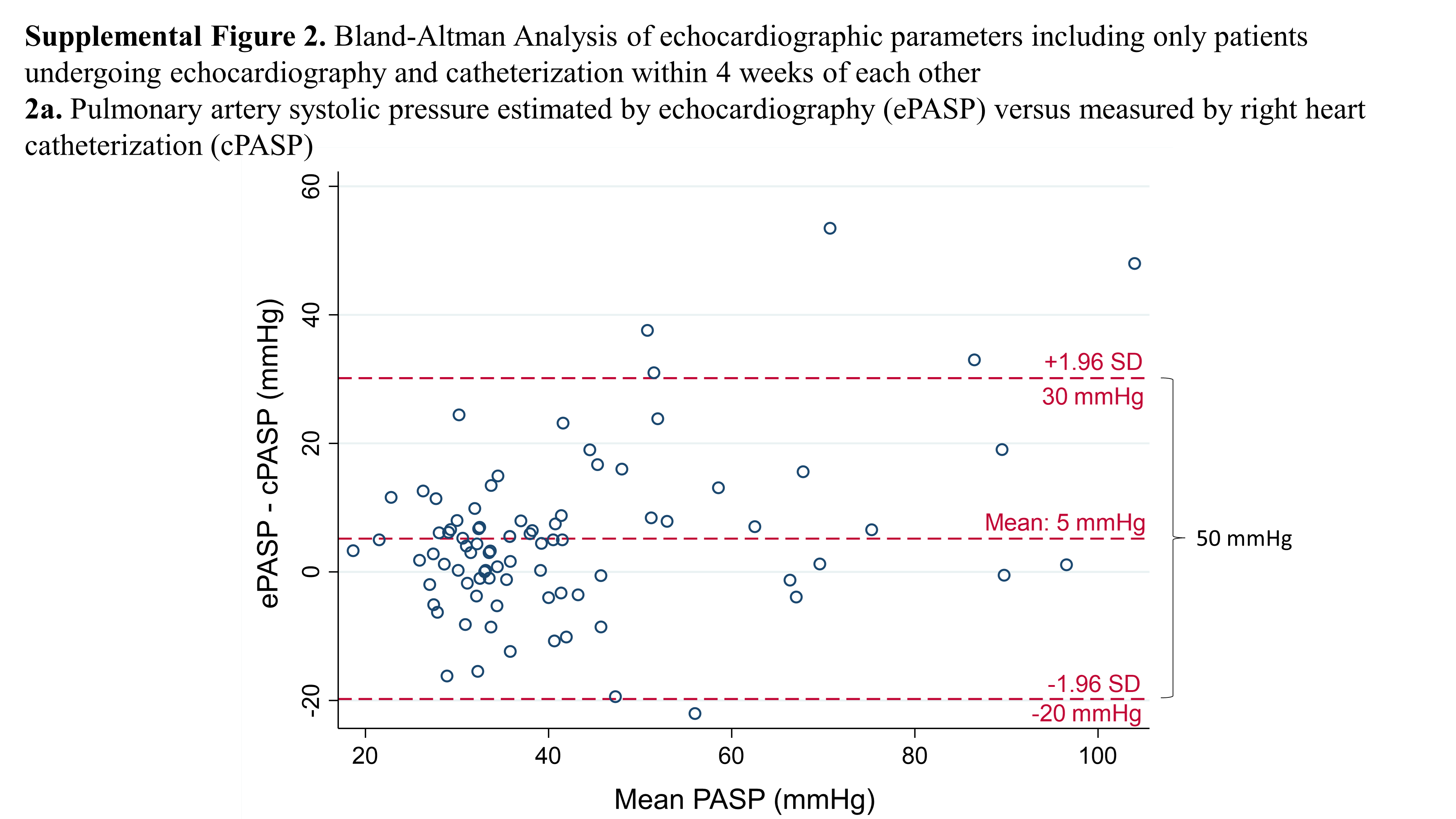

Supplement: Supplementary file 3 — Supporting information [file PUL2-13-e12183-s005.tif]

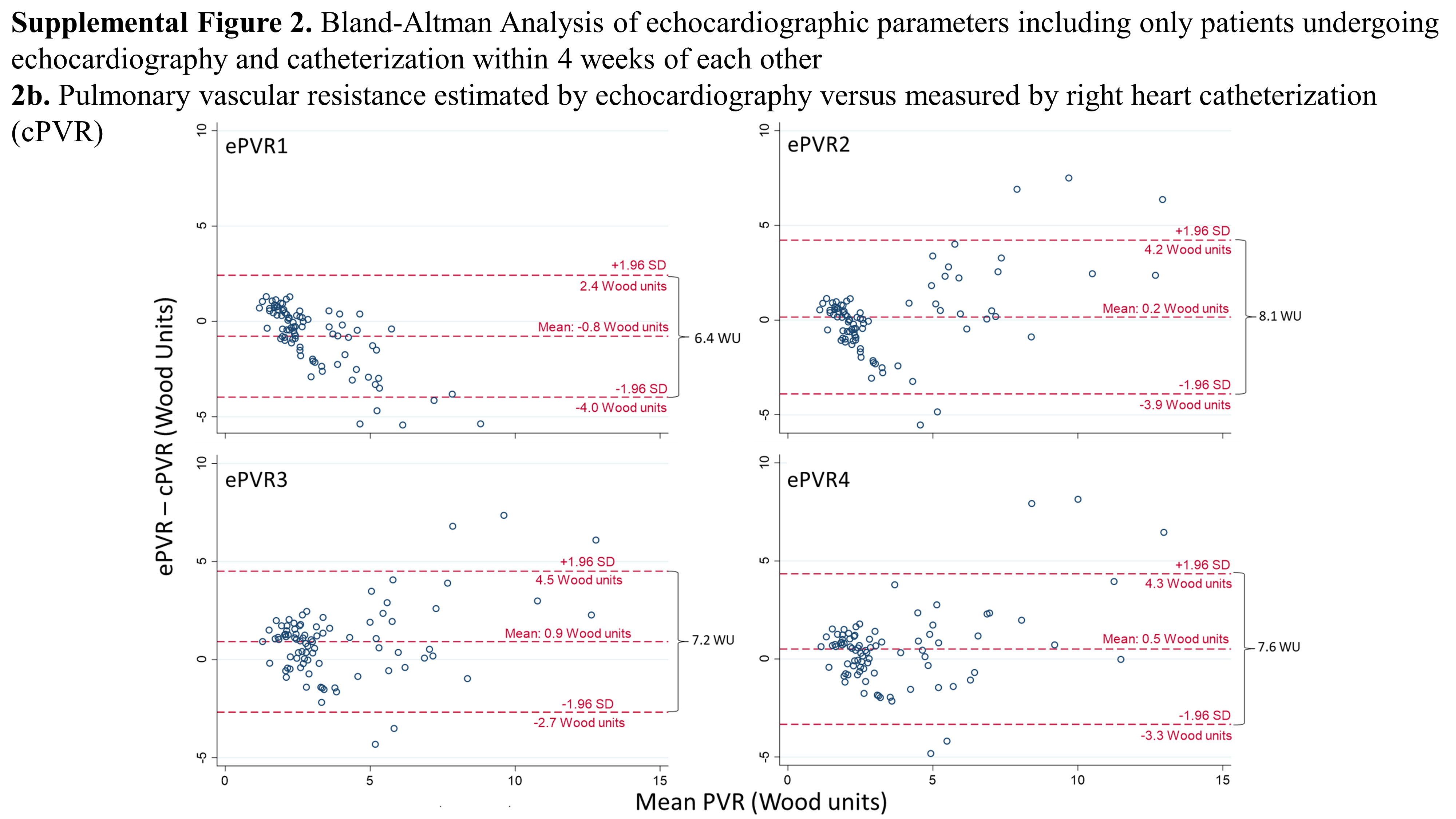

Supplement: Supplementary file 4 — Supporting information [file PUL2-13-e12183-s009.tif]

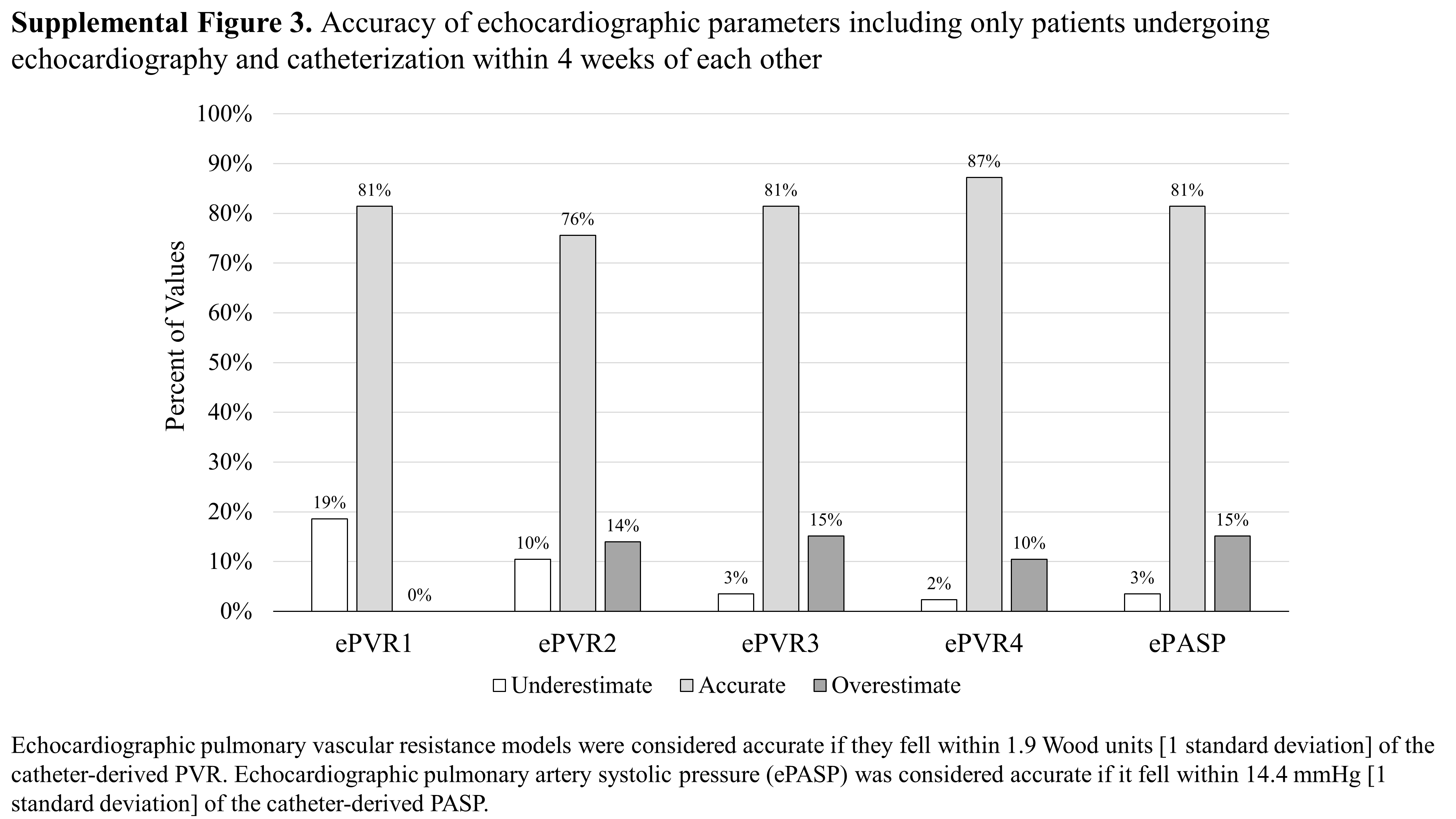

Supplement: Supplementary file 5 — Supporting information [file PUL2-13-e12183-s001.tif]

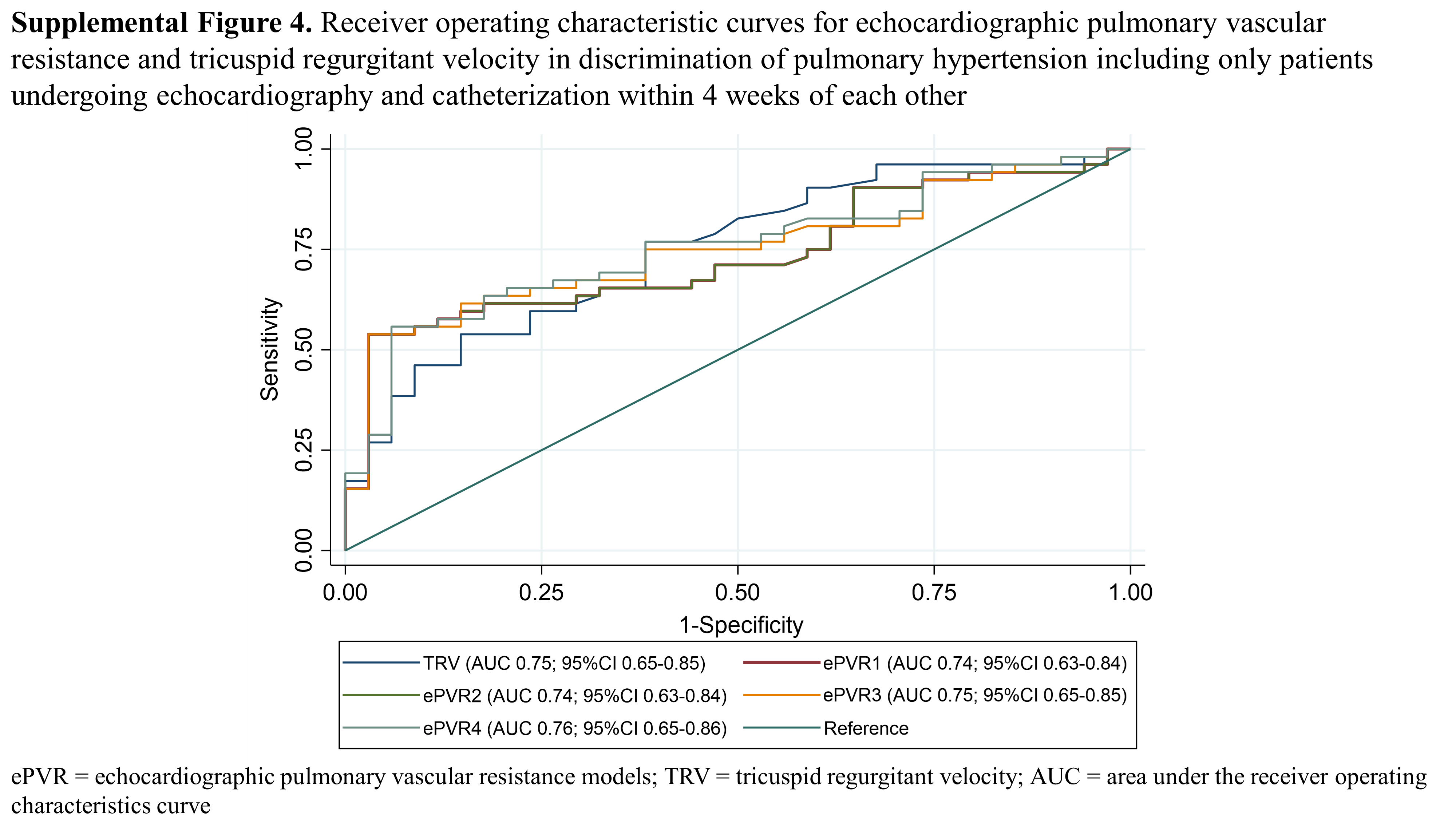

Supplement: Supplementary file 6 — Supporting information [file PUL2-13-e12183-s006.tif]

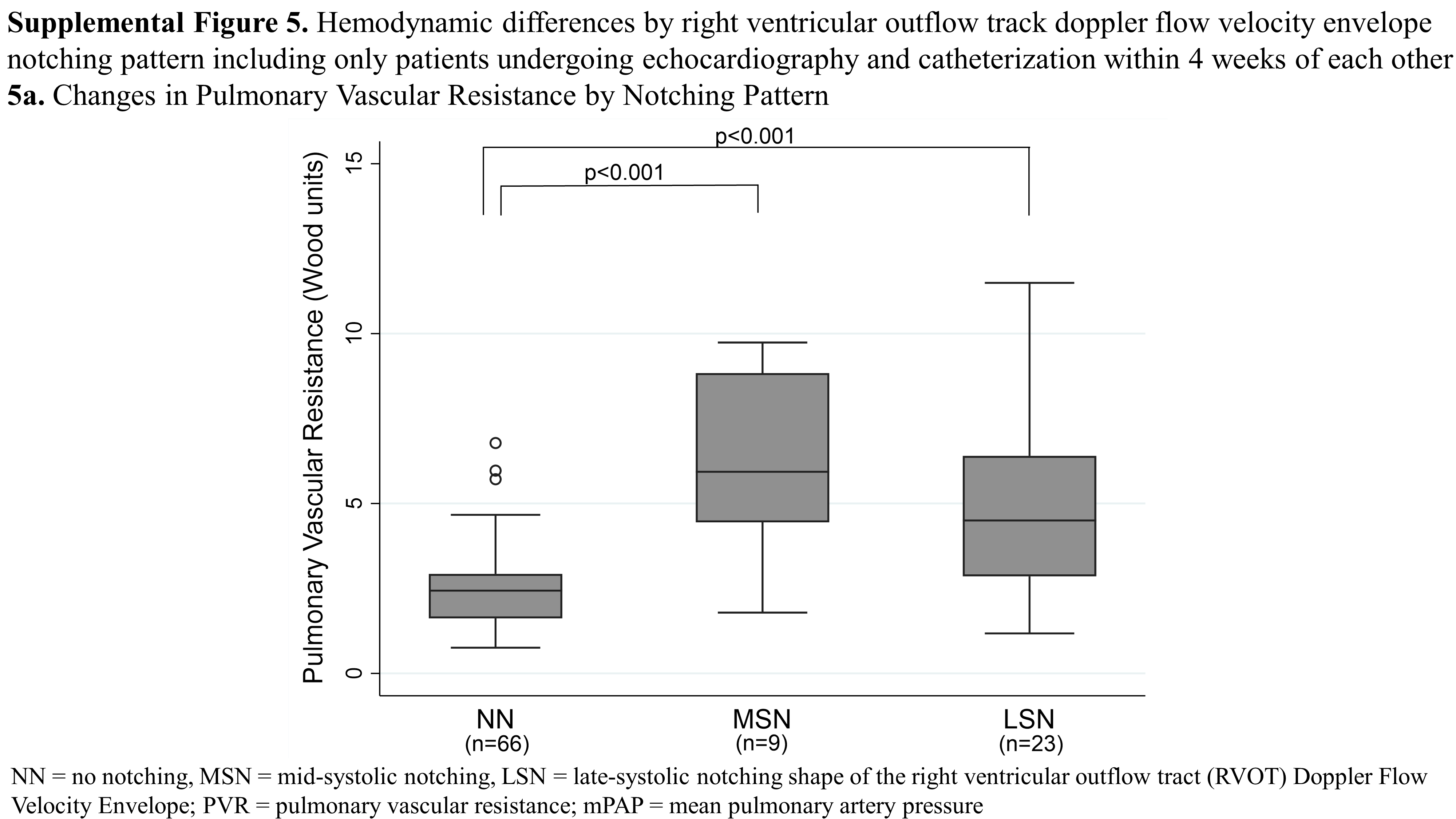

Supplement: Supplementary file 7 — Supporting information [file PUL2-13-e12183-s007.tif]

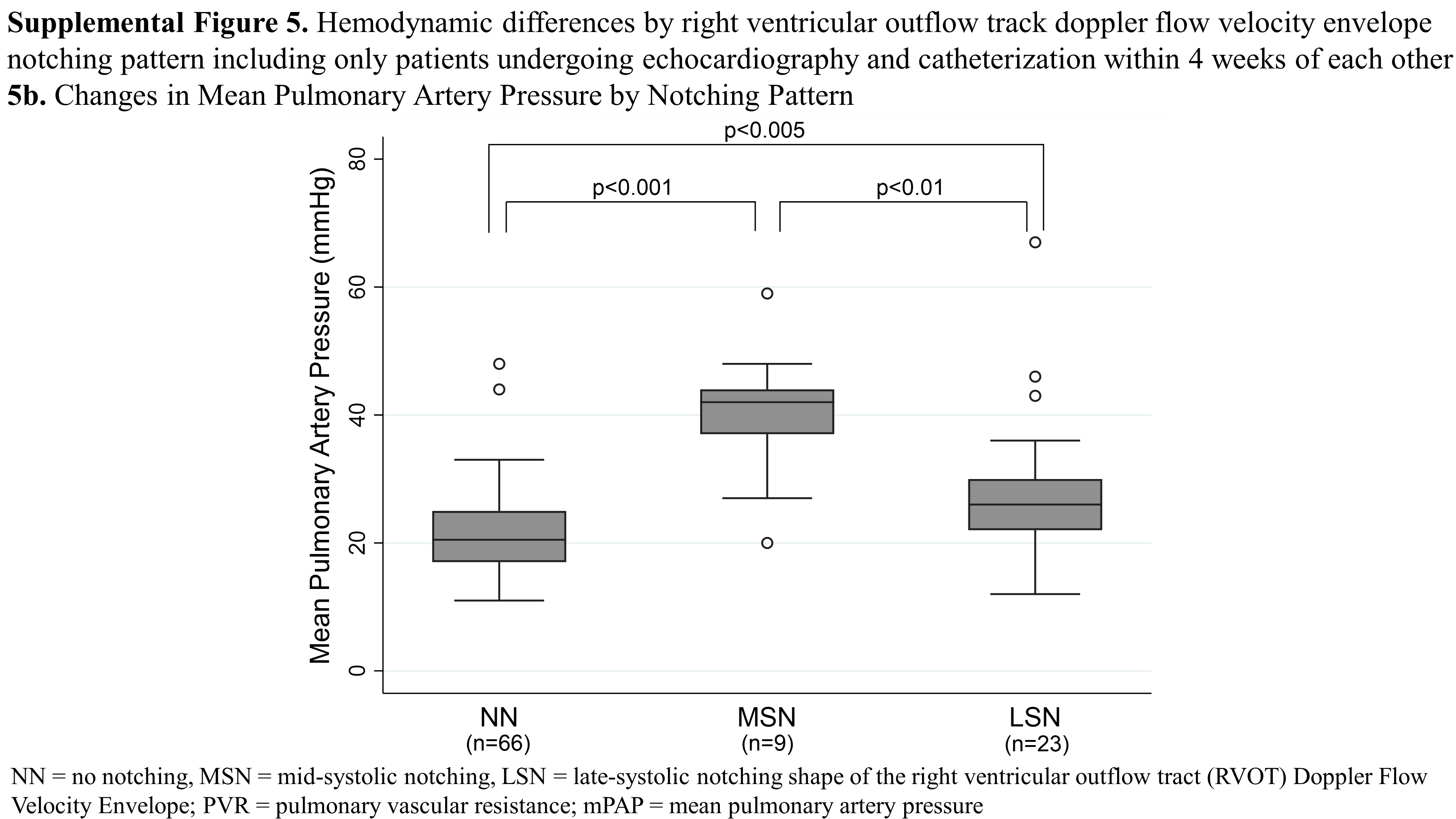

Supplement: Supplementary file 8 — Supporting information [file PUL2-13-e12183-s003.tif]
