# Supplementary material for: Echocardiographic estimation of pulmonary vascular resistance in advanced lung disease
Source: Pulm Circ. 2023 Jan 6;13(1):e12183. doi: 10.1002/pul2.12183 (PMC9817072; doi:10.1002/pul2.12183)
Supplement: Supplementary file 9 — Supporting information [file PUL2-13-e12183-s008.docx]

**Supplemental Table 1.** Testing characteristics for pulmonary hypertension screening

|  | **n** | **Sensitivity** | **Specificity** | **Correctly Classified** | **LR+** | **LR-** | **AUC (95% CI)** |
| --- | --- | --- | --- | --- | --- | --- | --- |
| TRV > 2.8 m/s | 133 | 73% | 65% | 70% | 2.07 | 0.41 | 0.69 (0.61-0.77) |
| MSN | 147 | 11% | 98% | 46% | 6.52 | 0.90 | 0.55 (0.51-0.58) |
| MSN or LSN | 147 | 40% | 84% | 58% | 2.61 | 0.70 | 0.62 (0.56-0.69) |
| ePVR1 > 2.0 Wood units | 133 | 72% | 51% | 64% | 1.47 | 0.55 | 0.61 (0.53-0.70) |
| ePVR1 > 3.0 Wood units | 133 | 39% | 94% | 60% | 6.63 | 0.65 | 0.67 (0.60-0.73) |
| ePVR2 > 2.0 Wood units | 133 | 67% | 69% | 68% | 2.14 | 0.48 | 0.68 (0.60-0.76) |
| ePVR2 > 3.0 Wood units | 133 | 40% | 92% | 60% | 5.13 | 0.65 | 0.66 (0.60-0.73) |
| ePVR3 > 2.0 Wood units | 133 | 95% | 16% | 65% | 1.13 | 0.31 | 0.55 (0.50-0.61) |
| ePVR3 > 3.0 Wood units | 133 | 76% | 65% | 71% | 2.14 | 0.38 | 0.70 (0.62-0.78) |
| ePVR4 >2.0 Wood units | 133 | 90% | 35% | 69% | 1.39 | 0.28 | 0.63 (0.55-0.70) |
| ePVR4 >3.0 Wood units | 133 | 60% | 84% | 69% | 3.81 | 0.48 | 0.72 (0.65-0.79) |

n=sample size; TRV = tricuspid regurgitant velocity; MSN = mid-systolic notching of the right ventricular outflow tract Doppler flow velocity envelope; LSN = late-systolic notching shape of the right ventricular outflow tract Doppler flow velocity envelope; ePVR = echocardiographic pulmonary vascular resistance model; LR+ = positive likelihood ratio; LR- = negative likelihood ratio; AUC = area under receiver operating characteristics curve; CI = confidence interval

**FIGURE CAPTIONS**
